# Passenger transport demand, fuel consumption, and emissions data for the Irish Passenger Transport Emissions and Mobility (IPTEM) model

**DOI:** 10.1016/j.dib.2022.108154

**Published:** 2022-04-11

**Authors:** Vera O'Riordan, Fionn Rogan, Tomás Mac Uidhir, Brian Ó Gallachóir, Hannah Daly

**Affiliations:** a Energy Policy and Modelling Group, MaREI, the SFI Research Centre for Energy, Climate and Marine, Environmental Research Institute, Cork, Ireland; b School of Engineering, University College Cork, Cork, Ireland

**Keywords:** Transport emissions modelling, CO_2_ emissions, Energy systems modelling, Occupancy, Public transport, Active modes

## Abstract

These data and analyses support the research article “How and Why We Travel – Mobility Demand and Emissions from Passenger Transport (O'Riordan et al., 2022). This article refers to a spreadsheet model, the Irish Passenger Transport Emissions and Mobility Model (IPTEM V2.9). The spreadsheet model is available for download from Zenodo (O'Riordan et al., 2022). The model and the underlying data, details the passenger transport demand by trip purpose (work, shopping, education etc.,), mode type (car, rail, bus, cycling, walking) and trip distance for Ireland over the period of 2009–2019. Passenger occupancy rates for public transport modes in Ireland, CO_2_ emissions intensities and annual CO_2_ emissions are also included in the Data in Brief. Assumptions and equations used to develop the IPTEM V2.9 are available in the Experimental design, materials, and methods section.

**Specifications Table**

**Table:** 

Subject	Engineering
Specific subject area	Passenger transport demand and CO_2_ emissions in Ireland
Type of data	Table Chart Figure
How the data were acquired	• National transport survey data gathered from Central statistics office (CSO) [3] • Information from the Irish Car Stock Model V 2.4 on an online repository [4] • Calculations as mentioned in the Data in Brief's reference paper and outlined in further detail in Experimental design, materials, and methods equations: Eqs. (1)–(10).
Data format	Raw Analyzed
Description of data collection	• Irish Car Stock Model, retrieved from an online repository [4], referred to in a Data in Brief [5] that provides technological stock data of car characteristics such as stock, mileage, and energy consumption per kilometre. • Population data made available from Eurostat [6] • National Travel Survey Data is available from the Central Statistics Office [3]
Data source location	Raw data sources used: National Travel Survey 2009, 2012, 2013, 2014, 2016 and 2019, available online at: https://www.cso.ie/en/statistics/ Sustainable Energy Authority of Ireland conversion rates, available online at: https://www.seai.ie/data-and-insights/seai-statistics/conversion-factors/ Irish Car Stock Model V2.4, available online at: https://zenodo.org/record/4651477#.YjD5fXrMKUk
Data accessibility	• Data is provided with this article in the following formats: Irish Passenger Transport Emissions and Mobility Model (IPTEM V2.9) is available on open-source repository Zenodo.Repository name: ZenodoData identification number: 10.5281/zenodo.6359991Direct URL to the data: 10.5281/zenodo.6359991
Related research article	V. O'Riordan, F. Rogan, B. Ó Gallachóir, T. Mac Uidhir, H. Daly, How and why we travel – Mobility demand and emissions from passenger transport, Transp. Res. Part D Transp. Environ. 104 (2022). 10.1016/j.trd.2022.103195.

**Value of the Data**

• This data provides clarity behind the modelling assumptions and methods used to model passenger transport demand and emissions in Ireland.
• While the data is Ireland specific, it serves as a guideline for the scientific community to ways to replicate similar modelling methods designed for other regions at a local, national, or international level.
• It provides valuable insights into the sources available at a national level which most European member states have freely available, and which can be used to replicate the modelling methods provided in the accompanying Transportation Research Part D article [1]. This data can be used to gain insights into the link between modal shift to low carbon dioxide passenger transport.
• Energy analysts can benefit from the detailed passenger transport demand information, serving to aid in replication of transport emissions and demand analyses. Policymakers also benefit from the information on occupancy rates, CO_2_ emissions intensity and passenger transport demand listed.

## 1 Data Description

The dataset referred to in this article exists as an Excel spreadsheet available on Zenodo [2]. The spreadsheet model has a series of sheets contained within the Excel file ‘IPTEM V2.9’. The dataset within this article provides secondary data from the Irish National Travel Survey conducted by the Central Statistics Office [7], data from the Irish Car Stock Model [4,8], energy and emissions conversion factors from the Sustainable Energy Authority of Ireland [9]. Occupancy, annual energy consumption figures, and passenger numbers from public transport providers: Dublin Bus (city bus transportation system) [10], Irish Rail (national rail network in Ireland) [11], Bus Éireann (national bus network in Ireland) [12], and Luas (light rail system in Dublin, Ireland) [13], are also included. Assumptions underpinning the IPTEM V2.9 model are also shared in the sheet “PKM (Passenger Kilometre) Calculation Assumptions”. Calculations for passenger kilometre demand in Ireland by trip purpose, trip distance and mode type are found in “PKM distance by distance and mode”, “PKM by distance and journey type” sheets. Excel formulae and references to previous sheets are embedded in the calculation sheets. Calculations for CO_2_ emissions by trip distance, mode type and trip purpose are found in Excel sheet “CO_2_ Emissions Intensity” and “CO_2_ Emissions by mode, purpose and distance”. An index and content description of the sheets in the IPTEM V2.9 model is listed in Table 1. This Data in Brief contains sample rows and entry values to describe the tables in the repository. The sample table entries are included in the data in brief to assist navigation of the accompanying Excel spreadsheet repository.

**Table 1:** Index of IPTEM V2.9 Excel spreadsheets.

Sheet label	Content Description
Readme	Provides information on the contact details of the author, and the latest revision date.
CSO Tables	Contains secondary data from the Central Statistics Office's National Travel Surveys in 2009, 2012, 2013, 2014, 2016 and 2019. Data is referred to in Table 3 - Table 26.
Irish Car Stock Model V 2.4	Contains references to the Irish Car Stock Model, which was developed by Daly and Ó Gallachóir [8], [4]. Data is referred to in Table 27 - Table 34: On-road factors for diesel cars (factors), Source: Irish Car Stock Model, [1,13]
Occup., Energy Cons., Emission	Shorthand for “Occupancy, Energy Consumption and Emissions”. This contains information on occupancy rates, passenger kilometre estimates for public transport. Data is referred to in Tables 35–58.
PKM (passenger kilometres) calculation Assumptions	Contains calculations for assumptions underpinning the Irish Passenger Transport Emissions and Mobility (IPTEM) model. Data and calculation tables are referred to in Tables 59–67.
PKM by distance and mode	Shorthand for passenger kilometre by distance and mode. It is a calculation of total passenger kilometres by year, trip distance and mode type. A sample row with descriptions of each of the entry fields is noted in Table 68. Method to calculate the Total Passenger Kilometres is listed in Eq. (3)
PKM by distance and purpose	Shorthand for passenger kilometre by distance and trip purpose. A sample row with descriptions of each of the entry fields is noted in Table 69. Methods to calculate Total Passenger Kilometres is listed in Eq. (3).
Passenger Kilometre Tables	Contains tables derived from pivot tables from Table 68. For years unsurveyed by the National Travel Survey, the values for passenger kilometres are interpolated. The passenger kilometres by mode type, trip distance and trip purpose over the period of 2009–2019 are listed in Tables 71–73.
CO_2_ Emissions Intensity	Data is listed in Tables 48–56. Method to calculate the CO_2_ emissions intensity is listed in Eq. (9).
CO_2_ Emission by mode, purpose, dist.	Shorthand for “CO_2_ Emissions by mode, purpose and distance.” Data is listed in Table 74. Methods to calculate the emissions are listed in Eqs. (9) and (10).
Emissions tables	Contains calculations that are interpolations of Passenger Kilometres by trip distance, mode type and trip purpose derived from pivot tables from Table 74. Outputs are listed in Tables 75–77.

### 1.1 CSO tables

The CSO tables are based on the National Travel Survey conducted by the Central Statistics Office (CSO). The survey was conducted for Ireland and is based on travel diaries by respondents in 2009, 2012, 2013, 2014, 2016 and 2019. Data for the intervening years is interpolated. A description of the mode types available in the survey is listed in Table 2.

**Table 2:** Overview of transport modes referred to in the IPTEM model [1].

Mode	Description
Private Car – Driver	People travelling in a car as the main driver
Private Car – Passenger	People travelling in a car driven by another person
Walk	People walking, this is also categorized as an “active mode” of transport
Bus	People taking the bus, there are two main bus transit providers in Ireland, Dublin Bus, which operates urban driving style city routes in Dublin, Ireland, and Bus Éireann, which provides a mix of urban and intercity driving. Private bus transport is assumed to be negligible.
Cycle	Includes the use of both mechanical bikes and e-bikes for cycling and is also categorized as an “active mode” of transport
Rail/DART/Luas	This mode choice refers to the three rail providers in Ireland; Irish Rail - which operates long distance rail in Ireland, DART- the Dublin Area Rapid Transit, a commuter rail operating in the Greater Dublin area and Luas - a city light rail which operates in Dublin
Taxi/hackney	People travelling in a car operated by a registered taxi driver
Lorry/Motorcycle/Other	This mode includes lorries, motorcycles and any other mode choice not included in the preceding categories

**Table 3:** Percentage distribution of journey distance for all regions, 2009–2019 Source: CSO, National Travel Survey [7].

	2009	2012	2013	2014	2016	2019
	%	%	%	%	%	%
**<2 km**	22	22.3	21.1	18.8	25.5	28.6

**Table 4:** Percentage distribution of journeys by mode of travel and distance < 2km, 2009 - 2019, Source: CSO, National Travel Survey [7]

	2009	2012	2013	2014	2016	2019
	%	%	%	%	%	%
Private car - Driver	40	51.9	46.3	49.1	50.3	51.4

**Table 5:** Percentage distribution of journeys by mode of travel and distance 2–4 km, 2009–2019, Source: CSO, National Travel Survey [7].

	2009	2012	2013	2014	2016	2019
	%	%	%	%	%	%
Private car - Driver	63.4	69.5	67.4	65.6	66.1	66.1

**Table 6:** Percentage distribution of journeys by mode of travel and distance 4–6 km, 2009–2019, Source: CSO, National Travel Survey [7].

	2009	2012	2013	2014	2016	2019
	%	%	%	%	%	%
Private car - Driver	71.7	72.6	73.1	70.6	71.9	70.6

**Table 7:** Percentage distribution of journeys by mode of travel and distance 6–8 km, 2009–2019, Source: CSO, National Travel Survey [7].

	2009	2012	2013	2014	2016	2019
	%	%	%	%	%	%
Private car - Driver	71.7	73.1	73.9	75.4	75.6	70.6

**Table 8:** Percentage distribution of journeys by mode of travel and distance >8 km, 2009–2019, Source: CSO, National Travel Survey [7].

	2009	2012	2013	2014	2016	2019
	%	%	%	%	%	%
Private car - Driver	74.0	79.7	79.3	78.3	80.2	71.9

**Table 9:** Population, 2009–2019, Source: Eurostat [6].

2009	2012	2013	2014	2016	2019
	Population				
4,521,000	4,589,000	4,610,000	4,638,000	4,726,000	4,904,000

**Table 10:** Number of respondents, National Travel Survey, 2009–2019, Source: CSO, National Travel Survey [7].

2009	2012	2013	2014	2016	2019
Number of respondents					
7,221	14,759	14,759	10,382	11,027	8,400

**Table 11:** Total journeys per person per day, National Travel Survey, 2009–2019, Source: CSO, National Travel Survey [7].

	2009	2012	2013	2014	2016	2019
Number of journeys per day	2.43	1.88	1.88	1.74	1.78	3

**Table 12:** Total journeys per year total, National Travel Survey, 2009–2019, Source: CSO: National Travel Survey [7].

	2009	2012	2013	2014	2016	2019
Number of journeys	4,007,543,571	3,165,688,023	3,171,485,717	2,940,102,001	3,079,198,792	5,369,880,000

**Table 13:** Private vehicle activity (vehicle kilometres, vkm), based on the Irish Car Stock Model, 2001–2018, Source:

2009	2012	2013	2014	2016	2019
vkm					
32,873,713,915	29,535,056,173	31,821,299,194	32,323,075,433	36,171,159,701	36,195,324,272

**Table 14:** Average journey distance by mode (vehicle kilometres), Source: CSO, National Travel Survey [7].

	2009	2012	2013	2014	2016	2019
	vkm	vkm	vkm	vkm	vkm	vkm
Private car - driver	14	14.3	15.4	15.6	16.3	13.6

**Table 15:** Weighting factor for distance based on mode, 2009–2019, calculation based on Eq. (1).

	2009	2012	2013	2014	2016	2019
Private car - driver	1.00	1.00	1.00	1.00	1.00	1.00

**Table 16:** Percentage distribution of journeys by reason of travel and distance <2 km, 2009 – 2019, Source: CSO, National Travel Survey.

	2009	2012	2013	2014	2016	2019
	%	%	%	%	%	%
Work	13	13.6	16.4	15.2	17.4	14.2

**Table 17:** Percentage distribution of journeys by reason of travel and distance, 2–4 km, 2009–2019, Source: CSO, National Travel Survey [7].

	2009	2012	2013	2014	2016	2019
	%	%	%	%	%	%
Work	17.0	16.8	19.1	20.3	24.4	17.4

**Table 18:** Percentage distribution of journeys by reason of travel and distance 4–6 km, 2009–2019, Source: CSO, National Travel Survey.

	2009	2012	2013	2014	2016	2019
	%	%	%	%	%	%
Work	23.0	18.9	22.5	21.8	27.3	22.1

**Table 19:** Percentage distribution of journeys by reason of travel and distance 6–8 km, 2009–2019, Source: CSO, National Travel Survey [7].

	2009	2012	2013	2014	2016	2019
	%	%	%	%	%	%
Work	24.0	24.5	23.4	22.6	33.3	22.3

**Table 20:** Percentage distribution of journeys by reason of travel and distance >8 km, 2009–2019, Source: CSO, National Travel Survey [7]

	2009	2012	2013	2014	2016	2019
	%	%	%	%	%	%
Work	37	32	21.8	33.4	38.1	31.4

**Table 21:** Average journey distance by purpose of travel, 2009–2019 (kilometres), Source: CSO, National Travel Survey [7].

	2009
	km
Work	18

**Table 22:** Reason for car journeys by percentage, based on average values from Dublin and rest of county as specified in Eq. (2).

	2009	2012	2013	2014	2016	2019
	%	%	%	%	%	%
Work	24.4	24.4	24.4	25.2	28.8	21.3

**Table 23:** Reason for public transport journeys by percentage, based on average values from Dublin and rest of county as specified in Eq. (2).

Reason for public transport journeys by percentage	2009	2012	2013	2014	2016	2019
	%	%	%	%	%	%
Work	28.8	28.8	28.8	25.3	32.2	35.4

**Table 24:** Reason for walking/cycling journeys by percentage, based on average values from Dublin and rest of county as specified in Eq. (2).

	2009	2012	2013	2014	2016	2019
	%	%	%	%	%	%
Work	17.7	17.7	17.7	15.6	18.5	21.0

**Table 25:** Reason for Lorry/motorcycle/other journeys by percentage, based on average values from Dublin and rest of county as specified in Eq. (2).

	2009	2012	2013	2014	2016	2019
	%	%	%	%	%	%
Work	52.1	52.1	52.1	50.9	57.8	48.3

**Table 26:** Reason for journey (percentage), Source: Irish Car Stock Model [7].

Reason for journey by percentage %	2009	2012	2013	2014	2016	2019
	%	%	%	%	%	%
**Work**	25	23.0	24.8	25.0	29.3	23.6

Passenger kilometres, occupancy figures, energy, and CO_2_ emissions intensity per passenger kilometre serviced are determined for the following public transit authorities in Ireland

1. Bus Éireann – The intercity and nation-wide bus service in Ireland
2. Dublin Bus – The urban bus service operating in Ireland's largest city, Dublin.
3. Irish Rail/DART – The heavy rail cross country and commuter rail service operating in Ireland
4. Luas – The light rail service

Passenger kilometres by trip purpose was also calculated. The following trip purposes are covered in this study

• Work
• Education
• Shopping
• To eat or drink
• Other
• Entertainment/Leisure/Sports
• Personal Business
• Companion/Escort Journey
• Visit family/friends

Trip distance categories from the National Travel Survey were as follows:

• < 2 km
• 2–4 km
• 4–6 km
• 6–8 km
• >8 km

The average of each of the categories are used in calculating overall Passenger Kilometre demand. The figure for passenger kilometres for the >8 km category was calculated through calibration with the Irish Car Stock Model.

### 1.2 Irish car stock model V 2.4

The tables listed in the sheet “Irish Car Stock Model V2.4” are extracted from an open source model [4]. A Data in Brief article corresponding to elements of an earlier version of the Irish Car Stock Model is available [5]. The methodology behind the calculation of vehicle kilometres and fuel consumption of private vehicles in Ireland is based on a study on technology stock modelling of private cars in Ireland [14].

**Table 27:** Energy consumption of hybrid vehicles (Source: Irish Car Stock Model, [4,5].

Energy Consumptionof Hybrid Vehicles	Year	2001
Engine size: < 1300cc	MJ/100km	1.45
Engine size: 1300cc–1700cc	MJ/100km	1.45
Engine size: > 1700cc	MJ/100km	2.73
Diesel energy consumption	MJ/100km	1.87
Diesel Energy consumption	MJ/km	0.02
Share of Petrol based energy consumption	%	0.60
Share of Electricity based energy consumption	%	0.40
Electrical Energy consumption of a Hybrid	MJ/km	0.02
Total Energy consumption of a Hybrid	**MJ/KM**	**0.04**

**Table 28:** Energy Consumption of Plug in Hybrid (MJ/100km), Source: [5]

Energy Consumption Plug in Hybrid	MJ/100km	2001
Share of Diesel based energy consumption	%	40%
Share of Electricity based energy consumption	%	60%
Average Diesel Plug in Hybrid	(MJ/100km)	1.06
Diesel Plug in Hybrid	(MJ/km)	0.01
Electric	(MJ/km)	0.03
Total		0.04

**Table 29:** Energy Consumption of Electric cars (MJ/km), Source: Irish Car Stock Model, [4,15]).

Energy Consumption of Electric cars	2001–2018
	MJ/km
	0.04222222

**Table 30:** Energy Consumption of Hybrid vehicles (MJ/km), Source: [4]

	2001
	MJ/KM
Diesel Hybrid	0.0140
Electric Hybrid	0.0169
All	**0.0309**

**Table 31:** Energy Consumption of electric cars (kWh/km), Source: Irish Car Stock Model, [4,14].

Energy Consumption of Electric cars	2001–2018
	kWh/km
Electricity	0.152

**Table 32:** Energy Consumption of Plug-in Hybrids (kWh/km), Source: Irish Car Stock Model, [4,14].

Energy Consumption of Plug in Hybrids	2001–2018
	kWh/km
Diesel	0.0382364
Electricity	0.0912
**Total**	**0.1294364**

**Table 33:** Energy consumption of Hybrid cars (kWh/km).

	2001	2002	2003	2004	2005	2006	2007	2008	2009	2010	2011	2012	2013	2014	2015	2016	2017	2018
	kWh/km																	

Diesel	0.050	0.050	0.050	0.048	0.054	0.059	0.062	0.063	0.069	0.068	0.067	0.067	0.067	0.067	0.067	0.067	0.067	0.067

**Table 34:** On-road factors for diesel cars (factors), Source: Irish Car Stock Model, [4,16].

	2001	2002	2003	2004	2005	2006	2007	2008	2009	2010	2011	2012	2013	2014	2015	2016	2017	2018
On-road factor																		

	0.13	0.13	0.13	0.13	0.13	0.12	0.14	0.16	0.17	0.2	0.25	0.27	0.33	0.39	0.41	0.42	0.42	0.42

### 1.3 Occupancy, energy consumption and emissions

**Table 35:** Occupancy Public Transport, based on passenger kilometres (Table 34) and Vehicle kilometres (Table 39).

	2010	2011	2012	2013	2014	2015	2016	2017	2018
	Number of people								
Dublin Bus (Urban Bus)	27	21	21	27	31	31	20	20	34

**Table 36:** Passenger kilometres from Public Transport, based on passenger kilometres by mode (Table 71).

	2009	2010	2011	2012	2013	2014	2015	2016	2017	2018	2019
	pkm										

Dublin Bus (Urban Bus)		1,657,005,445	1,249,888,239	1,214,931,149	1,536,441,904	1,765,668,627	1,777,305,989	1,160,134,746	1,195,332,290	1,962,684,829

**Table 37:** Multiplication of number of passengers serviced (Table 38) by public transport vehicle kilometres (Table 39).

	2009	2010	2011	2012	2013	2014	2015	2016	2017	2018	2019
	Non-unit factor										

Dublin Bus (Urban Bus)		7.23369E+15	6.91291E+15	6.49912E+15	6.29944E+15	6.62682E+15	6.82974E+15	7.18256E+15	7.95758E+15	7.99628E+15

**Table 38:** Number of passengers serviced by each public transport type.

	2009	2010	2011	2012	2013	2014	2015	2016	2017	2018	2019
	Number of passengers										

Dublin Bus (Urban Bus)		117,050,000	115,050,000	113,280,000	112,490,000	116,260,000	119,820,000	125,350,000	136,260,000	140,040,000

**Table 39:** Vehicle kilometres by public transport provider.

Vehicle kilometres	2009	2010	2011	2012	2013	2014	2015	2016	2017	2018	2019
	vkm										

Dublin Bus (Urban Bus)		61,800,000	60,086,140	57,372,160	56,000,000	57,000,000	57,000,000	57,300,000	58,400,000	57,100,000

**Table 40:** Diesel consumption per year Public Transport (kWh).

	2009	2010	2011	2012	2013	2014	2015	2016	2017	2018	2019
	kWh										

Dublin Bus (Urban Bus)		318,229,000	305,663,000	284,767,000	278,385,000	279,911,000	280,847,000	278,405,000	280,716,000	267,592,000

**Table 41:** Electricity consumption per year Public Transport (kWh).

	2009	2010	2011	2012	2013	2014	2015	2016	2017	2018	2019
	kWh										

Dublin Bus (Urban Bus)		6,500,000	6,422,000	5,786,000	5,430,000	5,240,000	5,223,000	4,614,000	4,517,000	4,457,000

**Table 42:** Natural Gas consumption per year Public Transport (kWh).

	2009	2010	2011	2012	2013	2014	2015	2016	2017	2018	2019
	kWh										

Dublin Bus (Urban Bus)		11,850,000	8,791,000	8,900,000	9,229,000	8,563,000	10,508,000	11,489,000	9,997,000	11,111,000

**Table 43:** CO_2_ Emissions Intensity by fuel type (gCO_2_/kWh) based on conversion factors from the Sustainable Energy Authority of Ireland [9].

	2009	2010	2011	2012	2013	2014	2015	2016	2017	2018
	gCO_2_/kWh									
Diesel	263.9	263.9	263.9	263.9	263.9	263.9	263.9	263.9	263.9	263.9

**Table 44:** Energy Intensity per passenger kilometer by Fuel Type - Dublin Bus (kWh/km) calculated from fuel consumption (Table 40, Table 41, Table 42) And per passenger kilometres (Table 36).

	2009	2010	2011	2012	2013	2014	2015	2016	2017	2018
	kWh/km									
Diesel		0.1921	0.2446	0.2344	0.1812	0.1585	0.1580	0.2400	0.2348	0.1363

**Table 45:** Energy Intensity by Fuel Type - Bus Éireann(kWh/km) calculated from fuel consumption (Tables 40–42) and passenger kilometres (Table 36).

	2010	2011	2012	2013	2014	2015	2016	2017	2018
	kWh/km								
Diesel	0.2585	0.3262	0.3161	0.2422	0.2215	0.2234	0.3301	0.3182	0.1724

**Table 46:** Energy Intensity by Fuel Type – Irish Rail (kWh/km) calculated from fuel consumption (Tables 40–42) and passenger kilometres (Table 36).

	2009	2010	2011	2012	2013	2014	2015	2016	2017	2018
	kWh/km									
Diesel		0.191	0.201	0.197	0.194	0.180	0.178	0.183	0.191	0.197

**Table 47:** Energy Intensity by Fuel Type – Luas (kWh/km) calculated from fuel consumption (Tables 40–42) and passenger kilometres (Table 36).

	2010	2011	2012	2013	2014	2015	2016	2017	2018
	kWh/km								
Electricity	0.0639	0.0485	0.0496	0.0481	0.0434	0.0457	0.0515	0.0454	0.0424

**Table 48:** CO_2_ Emissions Intensity by Dublin Bus (gCO_2_/km) based on calculation of energy intensity per passenger kilometer (Table 44) and CO_2_ Emissions intensity by fuel type (Table 43).

	2010	2011	2012	2013	2014	2015	2016	2017	2018
	gCO_2_/km								
Diesel	50.68	64.54	61.86	47.82	41.84	41.70	63.33	61.98	35.98

**Table 49:** CO_2_ Emissions Intensity of Bus Éireann (gCO_2_/km) based on calculation of energy intensity per passenger kilometer (Table 45) and CO_2_ emissions intensity by fuel type (Table 56).

	2010	2011	2012	2013	2014	2015	2016	2017	2018
	gCO_2_/km								
Diesel	68.22	86.09	83.42	63.92	58.45	58.95	87.10	83.98	45.49

**Table 50:** CO_2_ Emissions Intensity of Irish Rail (gCO_2_/km) based on calculation of energy intensity per passenger kilometer (Table 46) and CO_2_ Emissions Intensity by fuel type (Table 43).

	2010	2011	2012	2013	2014	2015	2016	2017	2018
	gCO_2_/km								
Diesel	50.47	52.92	51.99	51.14	47.61	47.09	48.23	50.41	52.02

**Table 51:** CO_2_ Emissions Intensity of the Luas (gCO_2_/km) based on calculation of energy intensity per passenger kilometer (Table 47) and CO_2_ Emissions intensity by fuel type (Table 44).

	2010	2011	2012	2013	2014	2015	2016	2017	2018
	gCO_2_/km								
Electricity	40.63	25.71	24.24	25.49	20.30	20.78	23.92	21.81	18.51

**Table 52:** CO_2_ Emissions Intensity of private vehicles by fuel type (gCO_2_/km), source: Irish Car Stock Model, [4,14].

	2001	2002	2003	2004	2005	2006	2007	2008	2009	2010	2011	2012	2013	2014	2015	2016	2017	2018
	gCO_2_/km																	

Diesel	179.35	180.07	182.36	182.81	181.61	185.70	186.53	184.62	182.36	179.44	177.20	175.76	175.53	174.56	174.49	173.71	172.36	173.48

**Table 53:** CO_2_ Emissions Intensity of Electric vehicles by fuel type (gCO_2_/km) calculated from CO_2_ Emissions intensity of electricity (Table 43) and the energy intensity of electric vehicles (Table 31).

	2009	2010	2011	2012	2013	2014	2015	2016	2017	2018
	gCO_2_/km									
Electricity	96.58	96.58	80.53	74.33	80.56	71.01	69.14	70.66	73.02	66.36

Electric vehicles by fuel type (gCO_2_/km)

**Table 54:** CO_2_ Emissions Intensity of Plug in Hybrids by fuel type (gCO_2_/km) calculated from CO_2_ Emissions intensity of fuels (Table 43) and the energy intensity of Plug-in hybrid vehicles (Table 32).

	2009	2010	2011	2012	2013	2014	2015	2016	2017	2018
	gCO_2_/km									
Electricity	57.95	57.95	48.32	44.60	48.34	42.61	41.49	42.40	43.81	39.82

**Table 55:** CO_2_ Emissions Intensity of Hybrids by fuel type (gCO_2_/km) calculated from the CO_2_ Emissions intensity of fuels and the energy intensity of hybrid vehicles (Table 30).

	2009	2010	2011	2012	2013	2014	2015	2016	2017	2018
	gCO_2_/km									
Electricity	38.63	38.63	32.21	29.73	32.22	28.41	27.66	28.27	29.21	26.55

**Table 56:** CO_2_ Emissions Intensity of private vehicles by fuel type per passenger kilometer (gCO_2_/pkm) based on CO_2_ Emissions intensity per vehicle kilometer (Tables 52–55), and occupancy of private vehicles (Table 61).

	2009	2010	2011	2012	2013	2014	2015	2016	2017	2018
	gCO_2_/pkm									
Diesel	120.4	120.9	122.4	122.7	121.9	124.6	125.2	123.9	122.4	120.4

**Table 57:** Energy intensity of private vehicles by fuel type (kWh/pkm).

	2009	2010	2011	2012	2013	2014	2015	2016	2017	2018
	kWh/pkm									
Diesel	0.456	0.458	0.464	0.465	0.462	0.472	0.474	0.470	0.464	0.456

**Table 58:** Synthesized CO_2_ Emissions intensity by mode type (gCO_2_/pkm) based on the CO_2_ Emissions intensity of cars (Table 56) and public transport (Tables 48–51).

	2009	2010	2011	2012	2013	2014	2015	2016	2017	2018	2019
	gCO_2_/pkm										

Private car –Driver	121.7	122.2	123.2	123.3	122.8	124.1	124.1	122.5	121.2	119.5	119.5

### 1.4 Passenger kilometre calculation assumptionsa

**Table 59:** Average distance by kilometer grouping for mode types.

	2009	2012	2013	2014	2016	2019
	km	km	km	km	km	km
<2 km	1	1	1	1	1	1

**Table 60:** Private vehicle kilometres from the Car Stock Model.

	2009	2012	2013	2014	2016	2019
	vkm	vkm	vkm	vkm	vkm	vkm
	32,873,713,915	29,535,056,173	31,821,299,194	32,323,075,433	36,171,159,701	36,195,324,272

**Table 61:** Private vehicle occupancy rates.

Private Vehicle Occupancy	2009–2019
	People
Private Vehicle	1.49

**Table 62:** Private vehicle passenger kilometres from the Irish Car Stock Model [4], [8].

	2009
	pkm
Passenger Kilometres	48,981,833,733

**Table 63:** Average distance by kilometre grouping for trip purpose.

	2009	2012	2013	2014	2016	2019
	km	km	km	km	km	km
<2 km	1	1	1	1	1	1

**Table 64:** Adjustment factor for distance based on trip purpose.

	2009	2012	2013	2014	2016	2019
Work	1	1	1	1	1	1
Education	0.888888889	0.888888889	0.888888889	0.888888889	0.888888889	0.888888889

**Table 65:** Vehicle Kilometres by fuel type of private cars (km).

Fuel Type	2001
PETROL	20,379,511,457
DIESEL	4,721,440,677
HYBRID	-
PLUGIN	-
ELECTRIC	-
Private Vehicle	25,100,952,134

**Table 66:** Number of journeys by trip purpose, calculated by Table 22, reason of journeys by percentage and total journeys per year, Table 12.

	2009	2012	2013	2014	2016	2019
	Number of journeys					
Work	1,001,885,893	728,108,245	786,528,458	735,025,500	902,205,246	1,267,291,680

**Table 67:** Average distance of journeys by trip purpose, calculated by dividing Table 66: Number of journeys by trip purpose by Table 72: Interpolation of Passenger Kilometres by trip purpose.

Average distance of journeys by trip purpose	2009	2012	2013	2014	2016	2019
	%	%	%	%	%	%
Work	21.3	21.7	15.5	25.3	25.9	15.4

### 1.5 Passenger Kilometres by distance and mode

**Table 68:** Sample row entry from Passenger kilometres by trip distance and mode type with additional reference row for Table references within the Data in Brief.

Title	Value description	Unit	Value	Data in Brief reference
Passenger kilometres by trip distance and mode type	Year		2009	
	Trip distance	km	<2 km	
	Mode Type		Private car - Driver	
	Decimal distribution of journey distance for all regions, 2009–2019	%	22	Table 3
	Distribution of journeys by mode of travel and distance < 2 km, 2009–2019	%	40	Table 4
	Distribution of journeys by mode of travel and distance 2–4 km, 2009–2019	%	0	Table 5
	Distribution of journeys by mode of travel and distance 4–6 km, 2009–2019	%	0	Table 6
	Distribution of journeys by mode of travel and distance 6–8 km, 2009–2019	%	0	Table 7
	Distribution of journeys by mode of travel and distance >8 km, 2009–2019	%	0	Table 8
	Average Journey Length	km	1.00	Table 14
	Mode distance adjustment factor		1.000	Table 15
	Number of journeys		4,007,543,571	Table 12
	Total Passenger Kilometres for the journey grouping	pkm	352,663,834	Calculation based on Eq. (4).
	Total Vehicle Kilometres (for that year)	vkm	32,873,713,915	Table 13

### 1.6 Passenger kilometres by distance and purpose

**Table 69:** Sample entry row passenger kilometres by trip distance and purpose with additional reference row for Table references within the Data in Brief.

Title	Value description	Unit	Value	Data in Brief reference
Passenger kilometres by distance and trip purpose	Year		2009	
	Trip distance	km	<2 km	
	Trip purpose		Work	
	Distribution of journey distance for all regions, 2009–2019	%	22	Table 3
	Distribution of journeys by mode of travel and distance < 2 km, 2009–2019	%	13	Table 16
	Distribution of journeys by purpose of travel and distance 2–4 km, 2009–2019	%	0	Table 17
	Distribution of journeys by purpose of travel and distance 4–6 km, 2009–2019	%	0	Table 18
	Distribution of journeys by purpose of travel and distance 6–8 km, 2009–2019	%	0	Table 19
	Distribution of journeys by purpose of travel and distance >8 km, 2009–2019	%	0	Table 20
	Average Journey Length		1.00	Table 21
	Trip purpose distance adjustment factor		1.000	Table 64
	Number of journeys		4,007,543,571	Table 12
	Total Passenger Kilometres		114,615,746	Calculation based on Eq. (3).
	Total Vehicle Kilometres		32,873,713,915	Table 13

### 1.7 Emissions by trip mode, purpose, and distance

**Table 70:** Sample entry row of emissions by trip mode, purpose, and distance with additional reference row for Table references within the Data in Brief.

Title	Value description	Unit	Value	Data in Brief reference
Passenger kilometres by mode type, trip purpose and distance	Year	2009		
	Mode Type	Private car - Driver		
	Purpose	Work		
	Trip Distance	<2 km	Km	
	Passenger Kilometres for that mode type across all purpose	41,186,265,415	Pkm	Table 72
	Percentage of journeys by trip purpose	24	%	Table 22
	Passenger Kilometres by purpose and mode type	10,028,855,628	pkm	Eq. (3)
	Average distance based on mode type	1		
	Km	Table 59		
	Average distance based on trip purpose type	1		
	Km	Table 63		
	Average distance based on trip purpose and mode type	1		
	Km	Eq. (5)		
	Distance weighting	0.02108148		Eq. (1)
	Calibration factor	1		
	Passenger Kilometres by distance category, mode type and trip purpose	211423118.6	Pkm	
	% Pkm for that year by distance, mode type and trip purpose category	0.003464316	%	Eq. (6)
	CO_2_ Emissions intensity by mode type	121.68	gCO_2_/pkm	Table 58
	Emissions from category	25726686424	gCO_2_	Eq. (10)
	Emissions from category	0.025726686	MTCO_2_	

### 1.8 Passenger kilometre tables

**Table 71:** Interpolation of Passenger Kilometres by mode type (2009–2019).

	2009
Bus	2,807,733,663
Cycle	223,983,168
Lorry/Motorcycle/Other	5,875,413,924
Private car - Driver	41,186,265,415
Private car - Passenger	7,789,254,560
Rail/Dart/Luas	2,755,466,733
Taxi/hackney	-
Walk	157,552,740
Total	60,795,670,202

**Table 72:** Interpolation of Passenger Kilometres by trip purpose.

	2009
Companion / escort journey	4,912,442,902
Education	2,294,831,660
Entertainment / leisure / sports	3,875,062,196
Other	5,507,218,474
Personal business	4,968,776,942
Shopping	10,147,549,168
To eat or drink	736,414,184
Visit family / friends	7,027,564,286
Work	21,336,198,042
Total	60,806,057,855

**Table 73:** Interpolation of passenger kilometres by trip distance.

Year	2009
<2 km	1,281,662,701
2-4 km	3,844,988,102
4-6 km	6,408,313,503
6-8 km	8,971,638,904
8+ km	40,289,066,993
	60,795,670,202

### 1.9 Emissions intensity

**Table 74:** Sample row from Emissions Intensity Table, with Table references for each entry.

Title	Value description	Unit	Value	Data in Brief reference
Emissions intensity	Year		2010	
	Mass transit or vehicle		Mass transport	
	Mode		Irish Rail (Heavy Rail)	
	Fuel Type		Diesel	
	Vehicle Kilometres	vkm	15,950,000	Tables 60, 39
	Occupancy	vkm/pkm	152.8	Tables 35, 61
	Passenger Kilometres	km	2,436,460,870	Table 36,
	CO_2_ Emissions Intensity	gCO_2_/pkm	50.47378412	Tables 48–53
	Total CO_2_ Emissions	gCO_2_	122,977,400,000	Calculation based on Eq. (10)
	Total CO_2_ Emissions	MTCO_2_	0.12	
	Energy Intensity	kWh/100pkm	19.13	Tabled 45–47, 57, Eq. (10)
	Energy Consumption	kWh	466,000,000	Calculation based on Eq. (8)
	Energy Consumption (MWH)	MWh	466,000	

### 1.10 Emissions tables

**Table 75:** Total emissions by trip purpose (MTCO_2_).

	2009
Companion/Escort Journey	1.08
Education	0.21
Entertainment/leisure/sports	0.47
Other	0.21
Personal Business	0.40
Shopping	1.39
To eat or drink	0.08
Visit family/friends	0.67
Work	1.45
Grand Total	5.96

**Table 76:** Emissions by trip distance category (MTCO_2_).

	2009
<2 km	0.125635
2–4 km	0.376906
4–6 km	0.628176
6–8 km	0.879447
8+ km	3.949344

**Table 77:** Emissions by mode type (MTCO_2_).

	2009	2010	2011	2012	2013	2014	2015	2016	2017	2018	2019
Bus	0.00	0.05	0.11	0.16	0.16	0.16	0.16	0.16	0.16	0.16	0.16

## 2 Experimental Design, Materials and Methods

This section will discuss the methods used to acquire the secondary data and calculate the primary data used in the IPTEM V2.9 spreadsheet.

### 2.1 Acquisition of secondary data

This section outlines the steps required to acquire, process, and analyse the data referenced in this article.

The National Travel Survey conducted by the CSO [7] forms a key source of secondary data for the “CSO Tables” sheet in IPTEM V2.9. The data was extracted from the interactive data tool available from the Central Statistics Office [3].

The open-source Irish Car Stock Model formed the basis of on the secondary data included in the “Irish Car Stock Model V2.4” spreadsheet [4]. The Irish Car Stock Model as described in Daly and Ó Gallachóir develops a picture of private car energy demand in Ireland [8]. The study documents the method and data needed to create a bottom-up private vehicle technology stock for Ireland. A Data in Brief corresponding to the Irish Car Stock Model is available [5].

Figures for the energy consumption, vehicle kilometres and passenger numbers for public transport operators in Ireland were extracted manually from Annual Reports by Luas [13], Bus Éireann [12], Irish Rail [11], and Dublin Bus [10]. These figures were referred to in “Occupancy, Energy Consumption and Emissions” sheet.

### 2.2 Equations for primary data

In this section, equations for the calculation of the primary data listed in the IPTEM V2.9 model are defined. The equations are previously referenced in the Data Description section as the method used to calculate values in table entries.

The CSO distance categorization does not account for mode types that typically service distances on the shorter end of the distance grouping. Distance weighting factors based on mode are calculated by comparing average distance travelled by that mode with the “Private car – driver” mode as shown in (xxx).

Eq. (1): *Distance weighting*

$$Distance\;Weighting_{mode,\;year}=\;\frac{Average\;distance\;of\;all\;journeys_{mode,\;year}\;}{Average\;distance\;for\;private\;car-driver_{year}}$$

The distribution of journeys by distance is recorded by the National Travel Survey over the period of 2009–2019.

Average values for reasons of journeys by mode type are based on aggregate values from Dublin and the rest of the country. As approximately 1 in 4 people in Ireland are Dublin based, a weighting of 0.25 is given to the Dublin statistics, and a weighting of 0.75 is given to the rest-of-country figures. The equation for this is outlined below:

Eq. (2): *Average value for reason of journey by mode type*

$$\begin{array}{ccc} & & Average\;reason\;of\;journey\;by\;mode\;type\\ & & \;=0.25(\%\;Reason\;for\;journey\;by\;mode\;type_{Dublin})\\ & & \;\;+\;0.75(\%\;Reason\;for\;journey\;by\;mode\;type_{Rest\;of\;Country}) \end{array}$$

Total Passenger Kilometres for a given distance and mode category is calculated as a function of the share of journeys by distance and the share of journeys that are of that mode type, then applying the average distance by the kilometer grouping and applying weighting factors based on the mode type as calculated in Eq. (1).

Eq. (3): *Total Passenger Kilometres*

$$\begin{array}{ccc} & & Total\;Pkm\;by\;distance\;and\;mode\;category\\ & & \;=\%\;distribution\;of\;journey\;distance\times Percentage\;of\;journeys_{mode}\;\\ & & \;\times \;average\;distance\;by\;distance\;grouping_{mode}\\ & & \;\times \;weighting\;factor_{mode}\;\times number\;of\;journeys \end{array}$$

Passenger kilometres for the intermittent years that were not surveyed (2010, 2011, 2015, 2017, 2018) are interpolated.

To calculate total passenger kilometres by distance and trip purpose, the method used in Distance weighting factors based on trip mode are applied to adjust the average distance calculated to reflect the average journey lengths given for a given trip mode [1]. Weighting factors based on trip purpose are also applied and calculated by comparing the average distance of journeys by trip purpose

Passenger kilometres by trip purpose was calculated as shown in Eq. (4).

Eq. (4): *Passenger kilometres by trip purpose*

$$\begin{array}{ccc} & & Passenger\;kilometres\;by\;trip\;purpose\\ & & \;=percentage\;distribution\;of\;journey\;distance\\ & & \;\times \;percentage\;distribution\;of\;joruneys\;by\;trip\;purpose\;and\;distance\\ & & \;\times \;average\;distance\;by\;kilomtre\;grouping\;for\;trip\;purpose\\ & & \;\times \;adjustment\;factor\;based\;on\;trip\;purpose\;\times total\;number\;of\;journeys \end{array}$$

Weighting factors based on trip purpose are also applied to adjust the average distance calculated to capture the varying average journey lengths for certain trip purposes [1]. Only figures from 2009 are available as average distances based on trip purpose are given from the 2009 National Travel Survey.

Average distance based on trip purpose and mode type is based on the average distance based on trip purpose type (Table 21) and average distance based on mode type (Table 59). As the average distance based on trip purpose is only calculated from the 2009 National Travel Survey, these figures are used for all journeys up to 2019.

Eq. (5): *Average distance based on trip purpose and mode type*

$$\begin{array}{ccc} & & Average\;distance\;based\;on\;trip\;purpose\;and\;mode\;type\\ & & \;=\;\frac{Average\;distance_{trip\;purpose}+Average\;distance_{mode\;type}}{2} \end{array}$$

The share of passenger kilometres for that year by distance, mode type and trip purpose, as listed in Table 69: Sample entry row passenger kilometres by trip distance and purpose with additional reference row for Table references within the Data in Brief. The share is based on comparing the passenger kilometres from the listed mode type, trip purpose and distance entry to the entire passenger kilometres calculated for that year

Eq. (6): *Share of passenger kilometres by distance, mode and trip purpose*

$$\begin{array}{ccc} & & \%\;Pkmforthatyearbydistance,modeandtrippurpose\;\\ & & \;=\frac{Passengerkilometresbydistance,mode,trippurpose}{TotalPassengerKilometres} \end{array}$$

The energy intensity of each mode type per passenger kilometre (Pkm) was calculated as follows in Eq. (7).

Eq. (7): *Energy Intensity of mode type per passenger kilometre*

$$EnergyIntensity\left(\frac{kWh}{pkm}\right)=\;\frac{EnergyintensityperkWh_{f,t}\times Energyconsumptionperyear_{f,t}}{Pkm_{t}}$$

Where:

• *f* is the fuel type
• *t* represents the transit provider.

Energy consumption of a given journey type by mode, trip purpose or trip distance is calculated as a function of the mode type's energy intensity and the passenger kilometres serviced by the journey's passenger kilometres for the specified trip purpose, distance, and mode.

Eq. (8): *Energy Consumption of a given journey purpose, distance and mode*

$$\begin{array}{ccc} & & EnergyConsumption_{purpose,distance,mode}\\ & & \;=EnergyIntensity_{mode}\;\left(\frac{kWh}{Pkm}\right)\times PassengerKilometres_{purpose,distance,mode}\;\left(Pkm\right) \end{array}$$

The CO_2_ emissions intensity of each mode type per passenger kilometre (Pkm) was then calculated as follows:

Eq. (9): *CO_2_ Emissions Intensity per passenger kilometre by mode type*

$$\begin{array}{ccc} & & CO2\;EmissionsIntensity\;\left(\frac{gCO2}{pkm}\right)\\ & & \;=\;\frac{C02\;emissionsintensityperkWh_{f,t}\times Energyconsumptionperyear_{f,t}}{Pkm_{t}} \end{array}$$

Where:

• *f* is the fuel type
• *t* represents the transit provider.

CO_2_ emissions intensity per kWh were based on the Sustainable Energy Authority of Ireland conversion rates [9].

Annual energy consumption values for private vehicles were derived from the Irish Car Stock Model [4], annual energy consumption values for bus, heavy rail and light rail were derived from national public transport annual reports [10], [11], [12], [13].

Total CO_2_ emissions (Table 75) is calculated as a function of the passenger kilometres by the given mode type, trip purpose and trip distance and the CO_2_ emissions intensity of the given mode type, trip purpose and passenger kilometre category (Table 48, Table 49, Table 50, Table 51).

The equation for total CO_2_ emissions is outlined in Eq. (10).

Eq. (10): *Total Emissions by journey mode type, trip purpose or journey distance*

$$TotalCO2\;Emissions\;\left(gC02\right)=EmissionsIntensity\;\left(\frac{gCO2}{pkm}\right)\times PassengerKilometres\left(pkm\right)$$

## Ethics statements

The authors have no conflicts of interest to declare. Ethics approval was not required for this data in brief.

## CRediT authorship contribution statement

**Vera O'Riordan:** Conceptualization, Methodology, Data curation, Writing – original draft. **Fionn Rogan:** Supervision, Writing – review & editing. **Tomás Mac Uidhir:** Writing – review & editing. **Brian Ó Gallachóir:** . **Hannah Daly:** Supervision, Writing – review & editing.

## Declaration of Competing Interest

The authors declare that they have no known competing financial interests or personal relationships that could have appeared to influence the work reported in this paper.

## Data Availability

IPTEM V2.9 (Original data) (Zenodo).

## Appendix Supplementary materials

Image, application 1
